# Clip-anchored floss traction-assisted endoscopic submucosal dissection combined with endoscopic tunneling in treatment of long circumferential early esophageal carcinoma

**DOI:** 10.1055/a-2351-3077

**Published:** 2024-07-15

**Authors:** Wen-juan Ding, Xiang-rong Zhou, Fu-qiang Liu, Zhi-qiang Du, Wei-hui Liu

**Affiliations:** 1 546231 Department of Gastroenterology, The Peopleʼs Hospital of Jianyang City, Jianyang, China; 2 Department Gastroenterology and Hepatology, Sichuan Provincial Peopleʼs Hospital, School Of Medicine, University of Electronic Science and Technology of China, Chengdu, China

During endoscopic submucosal dissection (ESD) for large esophageal lesions, problems like lumen obstruction caused by distal contraction of the detached tissue, narrow submucosal space, and poor exposure of the submucosa often occur 1 2 3 . Although tunneling techniques can reportedly treat such lesions, they have not been able to resolve the issue of the dissected tissue clogging the lumen, and dissecting the ridges between the tunnels is challenging 4 5 . In this study, we used a tunneling technique combined with floss traction to improve the long circumferential ESD of early esophageal cancer ( Video 1 ).

Clip-anchored floss traction-assisted endoscopic submucosal dissection combined with endoscopic tunneling in the treatment of long circumferential early esophageal carcinoma.Video 1

A 64-year-old woman was treated with ESD for a circular early esophageal cancer 7 cm in length. ( Fig. 1 **a** ). Circumferential markings and incisions were made at the anal and oral sides of the lesion. The dental floss was fixed to the mucosal flap on the oral side with a clip to apply appropriate traction ( Fig. 1 **b** ). The submucosal fibers were stretched gently, and the submucosal space was thoroughly exposed; then, a wide tunnel was established from the oral side to the anal edge ( Fig. 1 **c** ). Continuous traction was applied by pulling the floss, and the dissected tissue clogging the lumen was avoided, hence the lesion was then efficiently dissected ( Fig. 1 **d** ). After the lesion was fully dissected, it showed a completely circumferential 8-cm surgical wound with a smooth surface and no muscle damage ( Fig. 1 **e** ). The specimen was easily extracted by simply pulling the floss ( Fig. 1 **f** ).

**Fig. 1:**
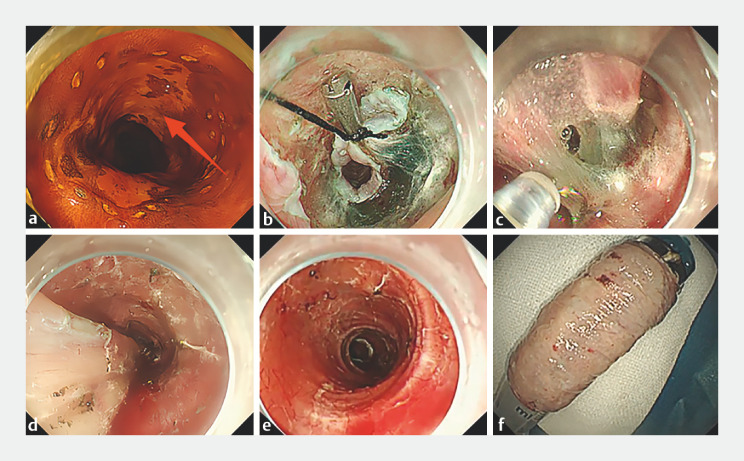
**a** A schematic of the clip-anchored floss traction-assisted endoscopic submucosal dissection combined with endoscopic tunneling in the treatment of long circumferential early esophageal carcinoma. Chromoendoscopy revealed an unstained area measuring 7 cm in length involving the esophageal circumference. **b** After circumferential incision, the dental floss was fixed to the mucosal flap on the oral side with a clip to apply appropriate traction. **c** Under continuous traction, the submucosal space was thoroughly exposed, and a wide tunnel was established from the oral side to the anal edge. **d** The submucosal fibers were gently pulled to prevent contraction of the resected tissue from blocking the surgical field of view and to dissect the lesion. **e** A completely circumferential 8-cm postoperative wound with smooth surface and no muscle damage was observed. **f** The en bloc-resected circumferential specimen was retrieved.

The tunneling technique combined with floss traction not only expands the space of the tunnel cavity but also avoids the obstruction of the surgical field of view by contraction of the dissected tissue. In addition, the tension provided significantly reduces the difficulty of dissecting the ridges between the tunnels. As the technology is simple to operate, it may be an attractive option for treating long circumferential early esophageal cancer.

Endoscopy_UCTN_Code_TTT_1AQ_2AD_3AZ

## Notes

Endoscopy E-Videos https://eref.thieme.de/e-videos
*E-Videos* is an open access online section of the journal *Endoscopy* , reporting on interesting cases and new techniques in gastroenterological endoscopy. All papers include a high-quality video and are published with a Creative Commons CC-BY license. Endoscopy E-Videos qualify for HINARI discounts and waivers and eligibility is automatically checked during the submission process. We grant 100% waivers to articles whose corresponding authors are based in Group A countries and 50% waivers to those who are based in Group B countries as classified by Research4Life (see: https://www.research4life.org/access/eligibility/ ). This section has its own submission website at https://mc.manuscriptcentral.com/e-videos .
